# Endoscopic ultrasound-guided glue embolization to prevent hemorrhage after accidental tract formation to the portal venous system during hepaticogastrostomy

**DOI:** 10.1055/a-2800-4753

**Published:** 2026-02-26

**Authors:** Chloë Hanssens, Elisabeth Dhondt, Pieter Hindryckx

**Affiliations:** 1 Department of Internal Medicine, University Hospital of Ghent, Ghent, Belgium; 2 549665 Department of Vascular and Interventional Radiology, University Hospital of Ghent, Ghent, Belgium; 3 60200 Department of Gastroenterology, University Hospital of Ghent, Ghent, Belgium

A 79-year-old woman was referred to our hospital for endoscopic ultrasound-guided hepaticogastrostomy (EUS-HGS). She suffered from malignant distal biliary obstruction in the context of metastasized pancreatic adenocarcinoma, leading to interruption of her chemotherapy. Classic endoscopic retrograde cholangiopancreatography was not possible due to an impassible duodenal stenosis for which she had previously undergone an EUS-guided gastroenterostomy.

**Fig. 1:**
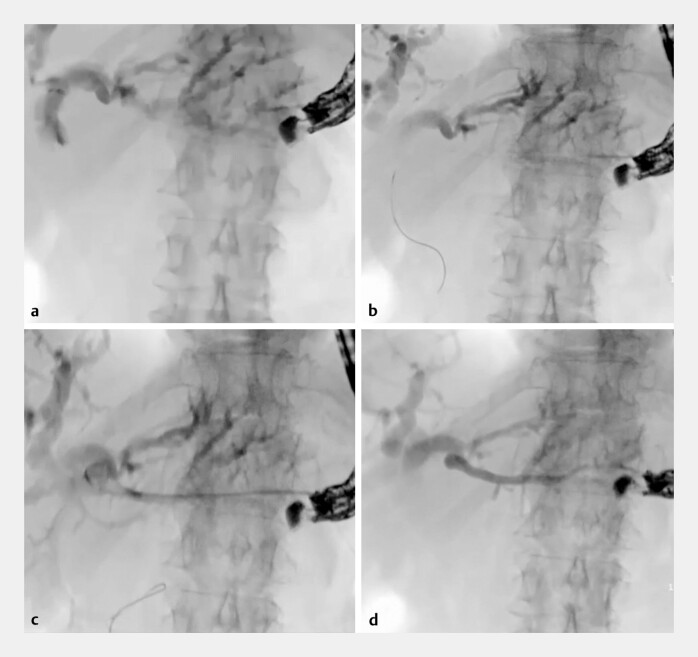
**a** Cholangiography after the puncture of a dilated left intrahepatic bile duct. **b** Passage of a guidewire through the needle into the direction of the liver hilum. **c** Opacification of a portal venous branch upon the injection of contrast through the cystotome. **d** Through-the-tome glue embolization of the portal venous branch to prevent hemorrhage upon withdrawal of the cystotome.

A linear ultrasound endoscope was introduced in the stomach ( Video 1 ). Fundic varices were noted but a safe trajectory was found to puncture a dilated intrahepatic bile duct with a 19-gauge needle (Expect slimline flexible needle, Boston Scientific). Adequate positioning of the needle tip was confirmed via bile aspiration and cholangiography ( Fig. 1 **a** ). The needle was flushed with aqua and a guidewire (0.025 angled tip VisiGlide2, Olympus) was introduced through the needle, following the expected course of bile ducts through the hilum towards the duodenum ( Fig. 1 **b** ). Over-the-wire dilation of the hepaticogastric tract was performed with a 6fr cystostome (Endo-flex). Contrast injection through the cystotome was performed prior to stent deployment. Surprisingly, this resulted in portography and not cholangiography ( Fig. 1 **c** ). We hypothesize that this could be either due to the (1) (millimetric) dislocation of the needle tip after cholangiography or (2) perforation of the guidewire through the bile duct wall into a venous blood vessel. Given the risk of severe hemorrhage upon withdrawal of the cystotome, the case was immediately discussed with an interventional radiologist (ED) who came on site. A decision for through-the-tome glue embolization was made. After flushing the cystotome with a glucose 10% solution, 4cc of a 50/50 mixture of histoacryl and lipiodol was injected, followed by a final flush with glucose 10% solution ( Fig. 1 **d** ). During injection, the cystotome was slowly withdrawn while monitoring adequate embolization on fluoroscopy. No bleeding or other adverse events occurred and the patient was discharged the next day. After informed consent was obtained, a second attempt of EUS-HGS was performed after 1 week with success. The cholestasis normalized and the patient could restart her chemotherapy.

This video shows the use of endoscopic ultrasound-guided glue embolization to prevent hemorrhage after the accidental formation of the tract between the upper stomach and the intrahepatic portal venous system during hepaticogastrostomy.Video 1

Haba et al. 1 have previously reported a similar case of accidental bougie dilation into a branch of the intrahepatic portal vein during EUS-HGS, managed with the EUS-guided placement of two embolizing coils.

Endoscopy_UCTN_Code_TTT_1AS_2AD
